# Integrin-Linked Kinase Plays an Active Role in the Regulation of Endothelial Senescence

**DOI:** 10.3390/cells15121081

**Published:** 2026-06-14

**Authors:** Wojciech M. Ciszewski, Ewa Macierzyńska-Piotrowska, Katarzyna Sobierajska

**Affiliations:** Department of Molecular Cell Mechanisms, Medical University of Lodz, Mazowiecka 6/8 Street, 92-215 Lodz, Poland; ewa.macierzynska-piotrowska@umed.lodz.pl (E.M.-P.); katarzyna.sobierajska@umed.lodz.pl (K.S.)

**Keywords:** endothelial cells, senescence, ILK, eNOS, rejuvenation, endothelial dysfunction, angiogenesis

## Abstract

**Highlights:**

**What are the main findings?**

- ILK is downregulated in replicative and stress-induced premature senescent endothelial cells.
- ILK silencing induced a senescence phenotype in endothelial cells, whereas ILK overexpression partially rejuvenated senescent endothelial cells.

**What are the implications of the main findings?**

- These findings propose a new mechanism of endothelial senescence cell regulation in an ILK-dependent manner.
- This study suggests that regulating ILK levels may restore the functionality of senescent endothelial cells and lead to effective vascular repair, thereby decreasing the prevalence of atherosclerosis and impaired angiogenesis during ageing.

**Abstract:**

Endothelial cells (ECs) play a critical role in physiological processes, including regulating blood fluidity, angiogenesis, and regulating the immune response. Integrins, which participate in sensing external stimuli and signal transduction, are crucial for the proper functioning of ECs. Like other cells, ECs undergo senescence, which is associated with their dysfunction and contributes to increased susceptibility to cardiovascular disease. However, the role of integrin-dependent pathways in endothelial senescence is poorly understood. Here, we identify integrin-linked kinase (ILK) as a crucial factor modulating endothelial function and senescence. Using two complementary models, replicative and stress-induced premature senescence, in endothelial cells of different origins, we show that the senescent endothelium shows phenotypic and functional dysfunction. Furthermore, we revealed that these modulations correlated with ILK downregulation. Functionally, ILK depletion in young ECs was sufficient to trigger a senescence-associated phenotype and manifested key features of endothelial dysfunction. In line with this, ILK restoration in senescent cells reduced selected senescence markers and improved endothelial function. Together, these findings show that ILK is not only correlated with endothelial ageing but also works as an active regulator of senescence-linked endothelial dysfunction. Thus, ILK, as a link between adhesion-dependent signalling and endothelial ageing, is a potential target for limiting age-associated vascular decline.

## 1. Introduction

The vascular endothelium is a versatile structure that separates circulating blood from underlying tissues. In addition to regulating and maintaining blood fluidity, it performs multiple regulatory functions [1]. The endothelium is a thin monolayer of endothelial cells that organises the growth and development of connective tissue cells that form the surrounding layers of the blood vessel wall. This process is controlled by paracrine/endocrine signalling that involves fibrinolytic, vasoactive, pro- and anticoagulant, and pro- and anti-inflammatory factors, as well as growth factors expressed by ECs [2]. Therefore, ECs must be able to receive numerous external stimuli, transmit signals to their environment, and interact with the extracellular matrix. Recent studies indicate a significant role of transmembrane proteins expressed by ECs, known as integrins, which act as receptors for many extracellular matrix (ECM) protein ligands [3]. Integrins link the extracellular environment to the intracellular cytoskeleton and regulate the propagation of signals to downstream kinases, such as integrin-linked kinase (ILK) [4].

Integrin-linked kinase (ILK) is a serine/threonine protein kinase known to bind directly to the cytoplasmic domains of β1 and β3 integrins and indirectly to actin through its interaction partners, including parvin, paxillin, and PINCH [5]. ILK is a multi-functional molecule that has major influence on the organisation of the actin cytoskeleton, microtubule dynamics, and regulation of centrosome clustering, thereby affecting cell spreading, migration, and cytokinesis [6]. Moreover, ILK regulates numerous diverse cellular processes, such as cell survival, proliferation, angiogenesis, cell adhesion, and invasion [7], and, as previously mentioned, plays an important role in various processes associated with cellular and life prolongation. Nevertheless, few studies have described a relationship between ILK and the senescence process, and its role in ageing remains poorly defined [8,9,10,11].

After a finite number of replication cycles, ECs, like other cell types, enter replicative senescence [12]: a permanent cell cycle arrest marked by reduced telomerase activity and telomere shortening. Different cellular stressors, such as oxidative stress induced by hydrogen peroxide (H_2_O_2_), have been demonstrated to trigger a similar phenotype, and this process is often referred to as stress-induced premature senescence (SIPS). Thus, more generally, senescence can be defined as a state of stable cell cycle arrest in response to diverse stresses [13]. Furthermore, senescent cells exhibit a characteristic phenotype that distinguishes them from other non-dividing cells, including growth arrest; dramatic changes in cell morphology, including increased cellular volume; upregulation of senescence-associated β-galactosidase (SA- β-galactosidase); and expression of p16 and p21 and secretion of soluble factors, known as the senescence-associated secretory phenotype [2]. ECs may additionally be characterised by decreased nitric oxide (NO) production, altered expression or phosphorylation of endothelial NO synthase (eNOS), increased expression of intercellular adhesion molecule-1 (ICAM-1) and plasminogen activator inhibitor-1 (PAI-1), or enhanced adhesiveness for monocytes [13]. All these changes in endothelial morphology and function can contribute to the development of many pathological disorders, such as vascular ageing and age-related vascular diseases, particularly atherosclerosis and hypertension [12].

Through this study, we provide insight into the role of ILK in the regulation of senescent ECs. We examined ILK protein levels during replicative and stress-induced senescence in endothelial cells. Furthermore, we investigated how modulation of ILK protein levels, through either silencing or overexpression, affects the senescent phenotype of endothelial cells and their functionality. Understanding the role of ILK in endothelial senescence is crucial to identifying the molecular pathways involved in endothelial pathophysiology.

## 2. Materials and Methods

### 2.1. Cell Lines

This study was performed using two endothelial cell models: Human Microvascular Endothelial Cells (HMEC-1), kindly provided by Kathryn Kellar from the Centers for Disease Control and Prevention, Atlanta, GA, USA, and primary Human Umbilical Vein Endothelial Cells (HUVECs), isolated in our laboratory according to the previously described procedure [14]. The use of human-derived HUVECs was approved by the Research Ethics Committee of the authors’ institution (approval No. RNN/261/15/KE), and all procedures followed the principles outlined in the Declaration of Helsinki. The cells were maintained in MCDB 131 medium supplemented with 10% (*v*/*v*) FBS, glutamine (2 mM), epidermal growth factor (EGF; 10 ng/mL), hydrocortisone (1 μg/mL; HMEC-1 only), and antibiotics (streptomycin—100 μg/mL, penicillin—100 units/mL) at 37 °C, in a humidified atmosphere with 5% CO_2_.

All cell cultures were routinely tested and confirmed to be mycoplasma-free. The experiments were conducted within passages 15–35 of HMEC-1 cells and 1–6 of HUVECs. For experiments comparing early and late endothelial passages, HMEC-1 cells at passage 15 and HUVECs at passage 1 were used as young controls, whereas passages 25 (medium) and 35 (late) for HMEC-1 cells and passage 5 for HUVECs represented senescence-associated stages, as specified in the relevant figure legends and Section 3. Media and supplements were obtained from Life Technologies (Paisley, UK), except for EGF and hydrocortisone, which were obtained from Sigma–Aldrich (Darmstadt, Germany).

### 2.2. Replicative-like Senescence

Replicative-like senescence was induced in endothelial cells in a state close to a permanent proliferation arrest. HMEC-1 cells stopped proliferating after 37–38 passages, whereas HUVECs stopped proliferating after 6–7 passages under our culture conditions.

HMEC-1 cells stored in our depot, frozen at passage 14, were thawed, reached subconfluency, seeded (passage #15), and either subjected to the appropriate treatment or to a growth and subculture cycle. After reaching passages 25 and 35, the cells were subjected to the appropriate treatment. In order to maintain the reproducibility of culture conditions and the number of passage cycles, HMEC-1 cells starting from passage 15 were sub-cultivated every 7 days at a ratio of 1:10, and the medium was renewed once a week (4 days after sub-cultivation). HUVECs freshly isolated in our lab were treated similarly: the time of sub-cultivation was 6 days at a ratio of 1:6, and the medium was renewed 3 days after sub-cultivation.

### 2.3. Stress-Induced Premature Senescence

Stress-induced premature senescence was stimulated using H_2_O_2 (_Sigma–Aldrich, Darmstadt, Germany), which is known to induce oxidative stress. Briefly, HMEC-1 cells at different passage numbers (#15, #25, and #35) were seeded in a 6-well plate and treated with 150 μM H_2_O_2_ for 2 h. Next, the medium was replaced, and the cells were cultured for 72 h, after which the H_2_O_2_ treatment was repeated. After another 72 h, cells were subjected to appropriate analysis. HUVECs at passages 1 and 5 were treated analogously, except that cell cultivation was performed 48 h after H_2_O_2_ treatment. H_2_O_2_ concentration and exposure time were selected based on preliminary experiments, in which these conditions reproducibly induced a senescence-associated phenotype while preserving sufficient cell viability for subsequent molecular and functional analyses. The different post-treatment culture times for HMEC-1 cells and HUVECs reflected differences in their growth characteristics and were chosen to allow for stable senescence-associated change in each model.

### 2.4. siRNA Transfection

A pooled set of four siRNAs targeting human ILK and a negative control siRNA (scramble) were employed (Dharmacon, Lafayette, CO, USA). The cells were transfected with siRNAs (50 nM) using Xfect™ Transfection Reagent (Clontech, Mountain View, CA, USA) according to the manufacturer’s protocol and silenced for 48 h. The silencing effect was confirmed each time by Western blot analysis.

### 2.5. Transient Plasmid Transfection

Endothelial cells (HMEC-1 cells at passage no. 33, and HUVECs at passage no. 4) were transfected with a vector containing ILK (pcDNA3.1-ILK, a generous gift from Dr. Paul McDonald, Cancer Research Institute, Vancouver, Canada) or the empty vector (pcDNA3.1, as a control) with Xfect™ Transfection Reagent (Clontech, Mountain View, CA, USA) according to the manufacturer’s instructions. Then, cells were cultured in medium supplemented with G418 (75 μg/mL) Sigma–Aldrich (Darmstadt, Germany), and after 1 (HUVEC) or 2 (HMEC-1) passages were subjected to an appropriate analysis. ILK overexpression was confirmed each time through Western blot analysis.

### 2.6. Proliferation Rate and Cell Morphology Analysis

Endothelial cells subjected to appropriate treatment were stained with 0.4% Trypan Blue solution (Life Technologies, Eugene, OR, USA) according to the manufacturer’s instructions. Next, the number of cells and their size (average cell size in microns) were quantified using a Countess™ II automated cell counter (Life Technologies, Bothell, WA, USA). The cell growth rate was calculated with the equation

$$growth\;rate=1/t\;\cdot \ln\left(N/N_{0}\right),$$

where *t* = hours of growth [h]; *N* = number of cells harvested; *N*_0_ = number of cells seeded.

### 2.7. SA-β-Galactosidase Activity Assay

A senescence-associated β-galactosidase activity assay was performed as described previously [15]. Briefly, cells grown on a 6-well microplate were pretreated with 100 nM bafilomycin A1 (Sigma-Aldrich, St. Louis, MO, USA) for 1 h in fresh cell culture medium, followed by 1 h incubation with 33 µM dodecanoylaminofluorescein di-β-D-galactopyranoside (C12FDG, Life Technologies, Eugene, OR, USA). Then, cells were washed with ice-cold PBS, harvested, resuspended in ice-cold PBS, and immediately analysed on a FACSCalibur (Becton–Dickinson, San Jose, CA, USA). A total of 20,000 events were analysed for each condition, and the results are shown as a percentage of positive SA- β-galactosidase cells on the dot plot.

### 2.8. Protein Extraction and Western Blotting

Protein extracts were prepared from endothelial cells grown on 6-well plates subjected to the appropriate treatment. Next, cells were lysed with M-PER Mammalian Protein Extraction Reagent (Thermo Scientific, Rockford, IL, USA) supplemented with cOmplete, Mini, EDTA-free Protease Inhibitor Cocktail (Roche, Mannheim, Germany), according to the manufacturer’s instructions. Then, supernatants were aliquoted and stored at −70 °C until used.

Western blotting was performed under the same conditions as described previously [16], and the primary antibodies were against vimentin (cat. nr: 5741), p16 (cat. nr: 18769), p21 (cat. nr: 2947), phospho-eNOS (Ser1177) (cat. nr: 9571), eNOS (cat. nr: 9586) (Cell Signalling Technology, Inc., Beverly, MA, USA), ILK (cat. nr: sc-20019), ICAM-1 (cat. nr: sc-390483), and GAPDH (Santa Cruz Biotechnology, Dallas, TX, USA). Band density was quantified using ImageJ software version 1.54 (Bethesda, MD, USA), and protein levels were normalised to GAPDH.

### 2.9. Tube Formation Assay

A capillary-like tube formation assay was carried out using 24-well plates pre-coated with Matrigel (Corning, Bedford, MA, USA), according to a previously described procedure with minor modifications [14]. Briefly, HMEC-1 cells and HUVECs were plated on the Matrigel surface at densities of 4 × 10^4^ and 2 × 10^4^ cells per well, respectively. After 8 h of incubation, tube-like structures were examined and documented using an Olympus microscope equipped with a digital camera attachment (Olympus Optical Co., Ltd., Tokyo, Japan), and the total tube length was measured in ImageJ software v. 1.54 using the Angiogenesis Analyzer plugin.

### 2.10. Fluorescence Microscopy

Cells were seeded onto sterile glass microscope slides (on Lab-Tek II chamber) and maintained at 37 °C under standard humidified culture conditions with 5% CO_2_. Following the indicated treatment, cultures were fixed in 4% formaldehyde prepared in PHEM buffer composed of 60 mM PIPES, 25 mM HEPES, 10 mM EGTA, and 4 mM MgCl_2_, pH 6.9. The buffer was supplemented with cOmplete™ EDTA-free Protease Inhibitor Cocktail, and subsequent labelling was performed according to the previously published protocol [17]. Fluorescence images were acquired using an EVOS FLoid Cell Imaging Station (Thermo Fisher Scientific, Bothell, WA, USA). Signal intensity was quantified in ImageJ v. 1.47 by measuring fluorescence within manually delineated regions of interest (ROIs).

### 2.11. Statistical Analysis

**Figure 1:**
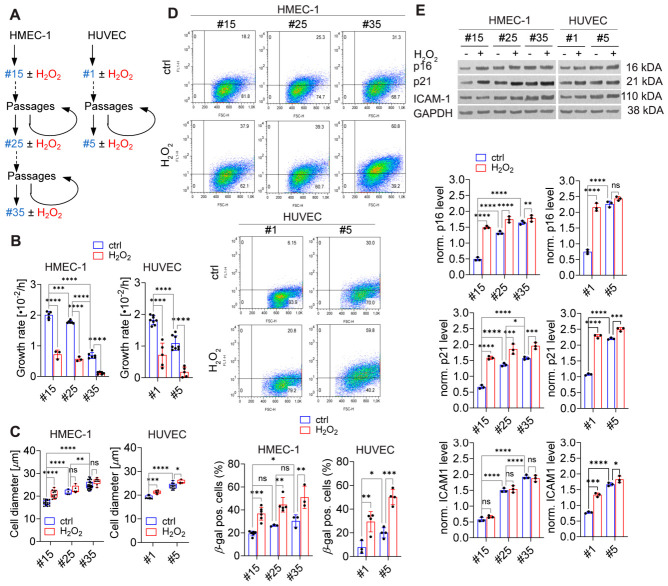
Characteristics of senescent endothelial cells (HMEC-1 and HUVEC). (**A**) Endothelial cells (ECs) in early and late passages were treated with H_2_O_2_ according to the experimental scheme. Then, (**B**) cell proliferation and (**C**) cell diameter were determined by Countess™ Automated Cell Counter. (**D**) FACS measured senescence-associated β-galactosidase activity. Representative dot plots are shown. (**E**) The level of senescence markers was analysed by Western blot. Representative blots are shown. The protein levels were normalised to GAPDH. The graphs display the means ± S.D. (*n* ≥ 3). ns—non-significant, * *p* < 0.05, ** *p* < 0.01, *** *p* < 0.001, **** *p* < 0.0001.

**Figure 2:**
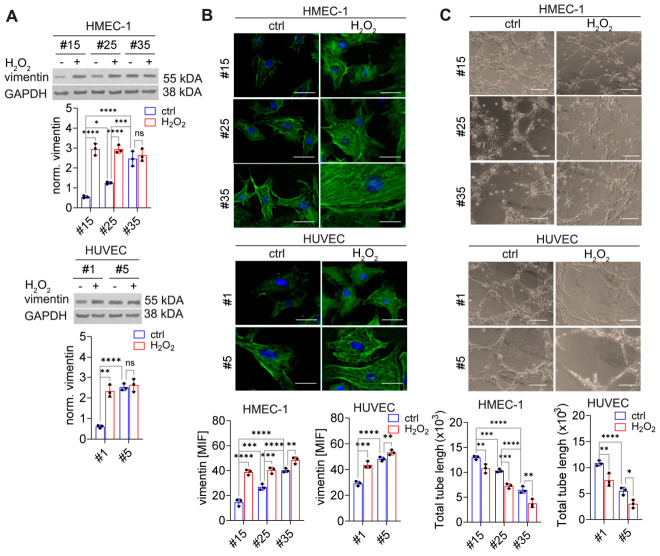
Senescence impairs endothelial cell function. Endothelial cells in early and late passages were treated with H_2_O_2_. Then, senescence-associated alterations of vimentin intermediate filaments were estimated by (**A**) Western blot and (**B**) immunostaining. Next, angiogenesis ability was studied by (**C**) capillary-like tube formation assay. Representative images are shown. Scale bars, 100 µm (immunostaining) and 50 µm (capillary-like tube assay). The graphs display the means of total tube length ± S.D. (*n* = 3). ns—non-significant, * *p* < 0.05, ** *p* < 0.01, *** *p* < 0.001, **** *p* < 0.0001.

**Figure 3:**
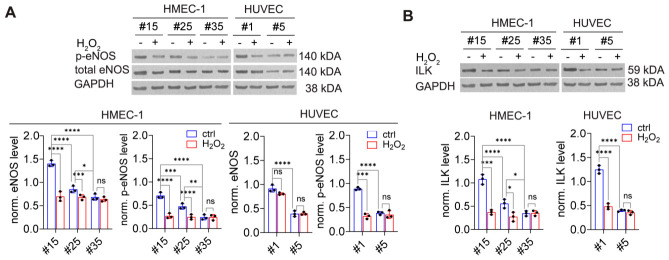
Integrin-linked kinase (ILK) is downregulated in both replicative and stress-induced premature senescent endothelial cells. Endothelial cells in early and late passages were treated with H_2_O_2_, and (**A**) eNOS activity and (**B**) ILK protein levels were analysed by Western blot. Representative blots are shown. The protein levels were normalised to GAPDH. The graphs display the means ± S.D. (*n* = 3). ns—non-significant, * *p* < 0.05, ** *p* < 0.01, *** *p* < 0.001, **** *p* < 0.0001.

**Figure 4:**
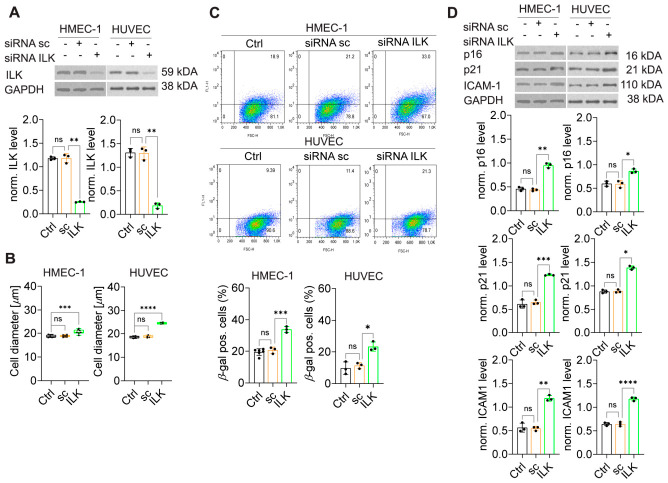
ILK silencing promotes a senescent phenotype and impairs endothelial cell function. Endothelial cells at low passages (HMEC-1 at passage no 15, and HUVEC at passage no 1) were transfected with siRNA ILK or scramble siRNA (negative control), and 48 h later, (**A**) the ILK silencing efficiency was analysed by Western blot. Then, the cells’ senescent phenotype was characterised. (**B**) Cell diameters were determined using a Countess™ Automated Cell Counter. (**C**) SA β-galactosidase activity was measured by FACS. Representative dot plots are shown. (**D**) The level of senescence markers was analysed by Western blot. Representative blots are shown. The protein level was normalised to GAPDH. The graphs display the means ± S.D. (*n* = 3). ns—non-significant, * *p* < 0.05, ** *p* < 0.01, *** *p* < 0.001, **** *p* < 0.0001.

**Figure 5:**
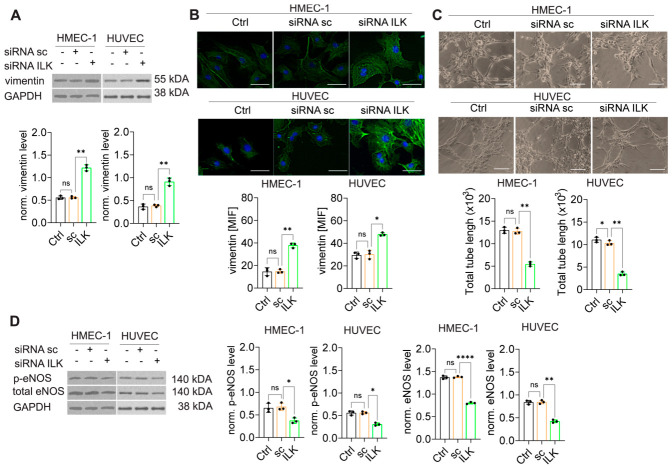
ILK silencing impairs endothelial cell function. Endothelial cells at low passages (HMEC-1 at passage no 15, and HUVEC at passage no 1) were transfected with siRNA ILK or scramble siRNA (negative control), and 48 h later, senescence-associated alterations of vimentin intermediate filaments were estimated by (**A**) Western blot and (**B**) immunostaining (Scale bars 100 µm). The effect of ILK silencing on angiogenesis ability was studied by (**C**) capillary-like tube formation assay (Scale bars 50 µm). Representative images are shown. (**D**) eNOS activity was analysed by Western blot. Representative blots are shown. The protein levels were normalised to GAPDH. The graphs display the means ± S.D. (*n* ≥ 3). ns—non-significant, * *p* < 0.05, ** *p* < 0.01, **** *p* < 0.0001.

**Figure 6:**
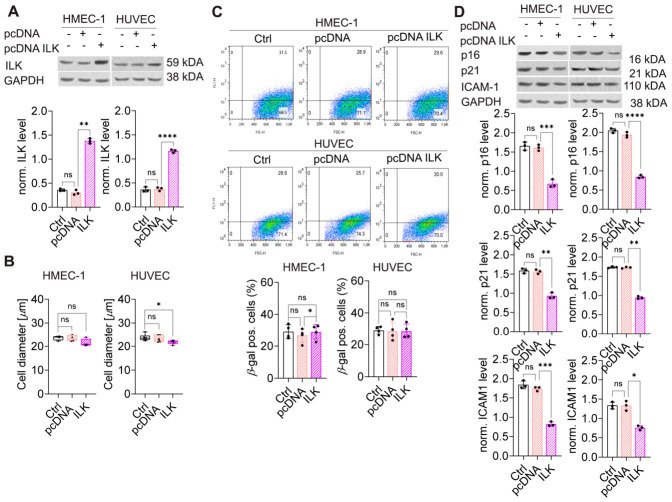
ILK overexpression partially attenuates senescence-associated changes in endothelial cells. Endothelial cells at late passage (HMEC-1 at passage no 33, and HUVEC at passage no 4) were transfected with pcDNA3.1-ILK or an empty pcDNA3.1 vector (as a control) and, after 2 passages, were subjected to analyses. (**A**) The ILK transient transfection efficiency was analysed by Western blot. Next, the cells’ senescent phenotype was characterised. (**B**) Cell diameters were determined using a Countess™ Automated Cell Counter. (**C**) SA β-galactosidase activity was measured by FACS. Representative dot plots are shown. (**D**) The level of senescence markers was analysed by Western blot. Representative blots are shown. The protein levels were normalised to GAPDH. The graphs display the means ± S.D. (*n* ≥ 3). ns—non-significant, * *p* < 0.05, ** *p* < 0.01, *** *p* < 0.001, **** *p* < 0.0001.

**Figure 7:**
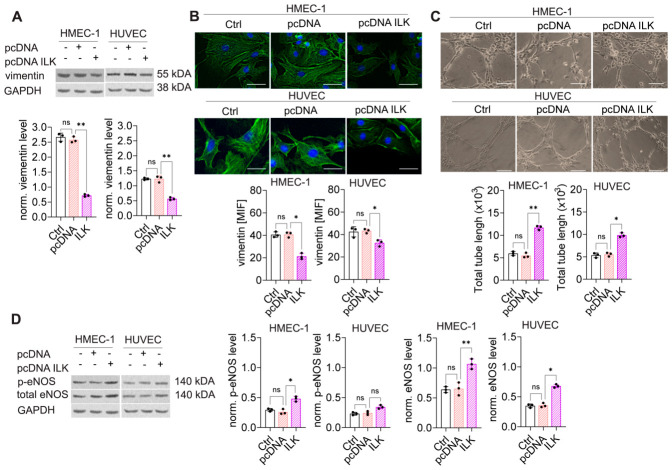
ILK overexpression restores functions of senescent endothelial cells. Endothelial cells at late passage (HMEC-1 at passage no 33, and HUVEC at passage no 4) were transfected with pcDNA3.1-ILK or an empty pcDNA3.1 vector (as a control) and, after 2 passages, were subjected to analyses. Senescence-associated alterations of vimentin intermediate filaments were estimated by (**A**) Western blot and (**B**) immunostaining (Scale bars 100 µm). The effect of ILK overexpression on angiogenesis ability was studied by (**C**) capillary-like tube formation assay (Scale bars 50 µm). Representative images are shown. (**D**) eNOS activity was analysed by Western blot. Representative blots are shown. The protein level was normalised to GAPDH. The graphs display the means ± S.D. (*n* ≥ 3). ns—non-significant, * *p* < 0.05, ** *p* < 0.01.

All data are expressed as the mean of at least three independent experiments and were tested for normality and homogeneity of variance. The statistical significance of the differences between the experiments was determined by two-way (Figure 1, Figure 2 and Figure 3) or one-way (Figure 4, Figure 5, Figure 6 and Figure 7) ANOVA followed by Tukey’s test, while the relationship between the variables was determined by linear regression analysis. All analyses were performed with the GraphPad Prism v. 10.6 software (GraphPad Inc., San Diego, CA, USA), and differences between means were considered significant at *p* ≤ 0.05.

## 3. Results

### 3.1. Establishment of Replicative and Stress-Induced Senescent Endothelium Culture Model

To investigate the role of ILK in endothelial senescence, we utilised two complementary models of cellular senescence: replicative senescence (RS) and stress-induced premature senescence (SIPS). Furthermore, we employed cells of different origins: Human Microvascular Endothelial Cell-1 (HMEC-1) and Human Umbilical Vein Endothelial Cells (HUVECs), derived from umbilical cord veins. HMEC-1 cells were originally established by transfection with the SV40 large T antigen, which should have immortalised them. However, during prolonged in vitro propagation, we observed changes in their morphology and phenotype, leading to proliferation inhibition after reaching 37–38 passages. Therefore, in our replicative senescence model, we decided to examine changes over time for passages 15, 25, and 35. HUVECs, which are primary endothelial cells, undergo a rapid replicative senescence process; therefore, their status was determined at passages 1 and 5. In parallel, endothelial cells were treated with hydrogen peroxide to provoke DNA damage and induce oxidative stress, both well-known hallmarks of stress-induced premature senescence (Figure 1A).

To validate the establishment of the senescent phenotype in the investigated cellular models, we examined a cardinal feature of senescent cells: their growth potential. We revealed that HMEC-1 cells and HUVECs undergoing replicative senescence decreased their growth rate by 2.9- and 1.7-fold, respectively, compared to the young cells (Figure 1B). Similarly, stress-induced senescence reduced growth by 2.8- and 2.5-fold in HMEC-1 cells and HUVECs, respectively. Another prominent characteristic of senescence is cellular enlargement. It has been proposed that such changes decrease the DNA-to-cytoplasm ratio, disrupting the cell proteome composition and, consequently, driving proliferation defects [18]. Therefore, we studied cell enlargement by estimating changes in cell diameter. We observed that cells undergoing RS exhibited an increase in cell size by 45.4% and 24.9% in HMEC-1 cells and HUVECs, respectively, whereas SIPS increased size by 22.3% and 11.5%, respectively (Figure 1C). The results showed that replicative senescence had twice the impact on cell enlargement as stress-induced senescence.

Next, we estimated the activity of the lysosomal hydrolase β-galactosidase (SA-β-gal) since its increase is associated with cell senescence [19]. We noticed an increase in SA-β-gal activity in ECs that underwent replicative senescence, which was 1.6- and 2.6-fold higher than in young HMEC-1 cells and HUVECs, respectively (Figure 1D). A similar effect was observed in SIPS cells, where SA-β-gal activity increased by 1.9- and 3.7-fold in HMEC-1 and HUVECs, respectively (Figure 1D).

During endothelial senescence, cells undergo changes associated with the appearance of characteristic protein markers, e.g., higher expression of p16INK4a, p21WAF1, and ICAM-1. Thus, in the next step, we examined the levels of these proteins in replicative and stress-induced senescent endothelial cells. We observed that cells undergoing RS increased p16 protein levels by 3-fold in both cell lines, and p21 protein levels increased by 2.4- and 2.1-fold in HMEC-1 cells and HUVECs, respectively (Figure 1E), while similar results were obtained for SIPS cells. Next, we analysed ICAM-1 levels and found increases of 3.3- and 2.2-fold in replicative senescent HMEC-1 cells and HUVECs, respectively (Figure 1E). In contrast, we observed only a slight increase in ICAM-1 levels in stress-induced senescence cells, at 1.2- and 1.7-fold in HMEC-1 cells and HUVECs, respectively.

### 3.2. Endothelial Cells Exhibit Impaired Angiogenic Function During Replicative and Stress-Induced Premature Senescence

An important function of ECs is their ability to form new vessel branches during angiogenesis. This process requires coordinated migration and cytoskeletal reorganisation of ECs in response to growth factor signalling, which is impaired during cellular senescence. Thus, we studied the senescence-associated alterations in cytoskeletal proteins, such as vimentin, whose levels change during senescence [20]. We observed an increase in vimentin protein levels in ECs undergoing replicative senescence, which were 4.5- and 4.1-fold higher than in young HMEC-1 cells and HUVECs, respectively (Figure 2A). A similar effect was observed in SIPS cells. We confirmed this observation by immunostaining experiments showing an increase in vimentin intermediate filaments in both senescence models, with the difference being that SIPS induced slightly greater changes (Figure 2B and Figure S1).

Next, we investigated the effect of the senescence process on ECs’ ability to promote angiogenesis through the capillary-like tube formation assay. RS caused a 50% reduction in total capillary length in both studied cell lines, and SIPS inhibited total capillary length by 17 and 30% in HMEC-1 cells and HUVECs, respectively (Figure 2C). The greatest effect on angiogenic impairment was observed when oxidative stress was induced in RS cells, reducing capillary length by 71% and 80% in HMEC-1 cells and HUVECs, respectively (Figure 2C).

### 3.3. eNOS Activity and Integrin-Linked Kinase Are Reduced in Senescent Endothelial Cells

Endothelial nitric oxide synthase (eNOS) is an enzyme predominantly expressed in the vascular endothelium that produces nitric oxide (NO), a critical regulator of blood vessel function [21]. However, eNOS activity and the production of NO are diminished in senescent endothelial cells [22]. Since we employed H_2_O_2_ for SIPS induction, which might directly or indirectly affect NO [23,24], we investigated eNOS status in our endothelial senescence cellular models, rather than direct secretion of NO. This revealed that RS decreased eNOS protein levels by 2.1- and 2.3-fold compared with those in young HMEC-1 cells and HUVECs, respectively (Figure 3A). Stress-induced senescence reduced eNOS at this same level in HMEC-1 cells, and only slightly in HUVECs. Since the synthesis of NO depends on eNOS activity, which is regulated by Ser1177 phosphorylation in response to various stimuli [25], we examined eNOS activity by determining the level of its phosphorylation. eNOS activity decreased by 2.9- and 2.3-fold in RS cells, compared with HMEC-1 cells and HUVECs, respectively, and by around 2.7-fold in SIPS cells in both cell lines (Figure 3A).

It has previously been shown that ILK plays an important role as a regulatory partner of eNOS, preventing eNOS uncoupling [26] and regulating its promoter [27]. Therefore, we investigated whether the reduction in eNOS protein levels and activity in senescent cells is accompanied by modulation of ILK protein levels. We observed a pronounced decrease in ILK protein levels in both cellular senescence models analysed. Replicative senescence resulted in a decrease in ILK, which was 3.1-fold lower than in young endothelial cells, and stress-induced senescence caused 2.9- and 2.6-fold decreases in ILK protein levels in HMEC-1 cells and HUVECs, respectively (Figure 3B).

### 3.4. Downregulation of ILK Induces Senescence-like Phenotype in Endothelial Cells and Drives Disability of Their Function

Since we demonstrated that ILK is downregulated during endothelial senescence, which correlated with decreased eNOS levels and activity, we next investigated whether ILK downregulation would stimulate a senescence-like phenotype in endothelial cells. We efficiently reduced ILK protein levels using siRNA targeting human ILK in endothelial cells. ILK protein levels were diminished by 79% and 86% in HMEC-1 and HUVEC, respectively (Figure 4A). ILK knockdown led to a change in cell size consistent with the senescence process. We observed that the diameter of HMEC-1 cells and HUVECs increased by 10% and 33%, respectively (Figure 4B). Furthermore, we observed an increase in SA-β-gal activity, which was 1.7- and 2.3-fold higher in ILK-silenced HMEC-1 cells and HUVECs, respectively (Figure 4C). Interestingly, we found that the protein levels of p16, p21 and ICAM-1, which are senescence markers, were also upregulated by the silencing of ILK (Figure 4D). ILK gene silencing resulted in approximately a 2- and 1.5-fold increase in the level of senescence marker proteins in HMEC-1 cells and HUVECs, respectively (Figure 4D).

Then, we investigated whether ILK downregulation would also affect endothelial cell behaviour and function. First, we studied changes in vimentin intermediate filaments of the cytoskeleton, which may translate into alterations in cell migration. We observed that ILK silencing caused an increase in vimentin protein levels by 2.2- and 2.4-fold in HMEC-1 cells and HUVECs, respectively (Figure 5A). Immunostaining experiments examining intermediate filaments showed a 2.6- and 1.6-fold increase in vimentin in ILK-silenced HMEC-1 and HUVECs, respectively (Figure 5B and Figure S2). Then, we investigated the role of ILK downregulation in angiogenesis, noticing that ILK silencing markedly inhibited angiogenesis, as assessed by capillary-like structure formation. The decline in ILK protein level caused a reduction in capillary length by 57 and 66% in HMEC-1 cells and HUVECs, respectively (Figure 5C).

We next examined the effect of ILK silencing on eNOS regulation in endothelial cells. Consistent with previous reports linking ILK to eNOS regulation, ILK downregulation resulted in decreased eNOS protein levels and activity, as measured by Ser1177 phosphorylation (Figure 5D). We revealed that eNOS protein levels decreased by 2- and 1.7-fold in ILK-silenced HMEC-1 cells and HUVECs, respectively. At the same time, eNOS activity decreased by 45% in both studied cell lines (Figure 5D).

### 3.5. Upregulation of ILK Partially Reverses Senescent Phenotype in Endothelial Cells and Improves Its Function

To confirm that ILK plays a critical role in modulating endothelial senescence, we tested whether increased ILK protein levels in senescent ECs would alter their senescence-associated phenotype and rejuvenate them. Replicative senescent endothelial cells were transfected with a vector containing ILK, which resulted in an increase in protein levels by 4.5- and 3.1-fold when compared with senescent HMEC-1 cells and HUVECs transfected with a control vector, respectively (Figure 6A). ILK overexpression had only a minor effect on cell size, decreasing cell diameter by less than 10% in both tested cell lines (Figure 6B), and had no effect on SA-β-gal activity (Figure 6C).

At the same time, we found that ILK-overexpressed senescent cells exhibited significant downregulation of senescence protein markers, such as p16, p21, and ICAM-1, compared to cells transfected with the control vector (Figure 6D). In general, ILK overexpression led to attenuation of the senescence-associated phenotype, as assessed by senescence markers, by 74% for p16 protein, 70% and 88% for p21 protein, and 64% and 81% for the ICAM-1 protein in HMEC-1 cells and HUVECs, respectively.

Hence, we demonstrated that increased ILK expression in cells undergoing replicative senescence could attenuate selected features of their senescence-associated phenotype; we asked whether ILK itself could have the potency to restore endothelial function. Similar to studies on the effects of ILK downregulation on cell function, we first tested whether ILK upregulation in senescent cells would affect the reorganisation of the vimentin cytoskeleton. We found that vimentin protein levels decreased by 3.6- and 2.2-fold in HMEC-1 cells and HUVECs transfected with a vector containing ILK, respectively (Figure 7A). We obtained similar convergent results for vimentin intermediate filaments, where the level was restored by 69% and 81% for HMEC-1 cells and HUVECs overexpressing ILK, respectively (Figure 7B and Figure S3). Importantly, overexpression of ILK in senescent endothelial cells restored the angiogenesis ability of HMEC-1 cells and HUVECs, as measured by the formation of capillary-like structures, by 100% and 90%, respectively (Figure 7C).

Consistent with the observed functional restoration, we also tested whether ILK upregulation in senescent cells would restore eNOS levels and activity. We observed that eNOS protein levels increased by 1.6- and 1.9-fold in ILK-overexpressing senescent HMEC-1 cells and HUVECs, respectively, compared with senescent cells transfected with a control vector (Figure 7D). eNOS activity was also increased by ILK overexpression in senescent cells, at 1.84- and 1.42-fold in HMEC-1 cells and HUVECs, respectively (Figure 7D). Taking into account the decreases in eNOS levels and activity in replicative senescent cells, it should be noted that ILK overexpression substantially improved eNOS levels (by 80%) and its activity (by 62%) in both tested cell lines.

## 4. Discussion

ECs form the endothelium, a single, semipermeable structural layer on the basement membrane that lines the inner surface of vessels and forms the boundary between the vessel wall and flowing blood. Its structural and functional integrity is essential for the proper functioning of the circulatory system, controlling the degree of vasodilation and vasoconstriction and thereby regulating regional blood flow [28]. However, the EC monolayer is not only a barrier between blood and tissues but also a specific endocrine organ. The endothelium is responsible for the extravasation of growth factors, hormones, fluids, macromolecules, and blood cells [29]. The endothelium also plays an important role in regulating the immune response and inflammation during defence against infection. ECs direct inflammatory cells, regulate platelet adhesion and aggregation, and stimulate leukocyte activation, adhesion, and transmigration [29]. Thus, ECs must be constantly poised to sense and respond to changes within their environment. A primary mechanism for such sensory perception is the utilisation of integrins. These bi-directional transmembrane signalling receptors mediate the attachment of cells to the extracellular matrix (ECM) and are involved in outside-in and inside-out signalling that promote endothelial cell motility and proliferation, or inhibit endothelial cell activities [30]. In endothelial cells, integrin activation is induced by mechanical stress and by growth factors, including VEGF, which is known as the “master switch” of angiogenesis [30]. Activated integrins function as docking modules for a plethora of intracellular signalling proteins, altogether known as the “adhesome” [31]. A prominent component of the integrin adhesome is the protein integrin-linked kinase (ILK).

Since the endothelium regulates many important functions, including the immune response, ECs are constantly subjected to multiple stimuli that may damage them, impair cellular homeostasis, and, consequently, lead to cellular senescence. It has been experimentally confirmed that vascular endothelial cell ageing occurs in vivo [32], which leads to changes in their phenotype and dysfunction, potentially contributing to the pathogenesis of age-related vascular diseases, such as impaired angiogenesis [33]. Generally, cellular senescence is defined as a persistent arrest of the cell cycle, accompanied by phenotypic changes in gene expression, morphology, and cellular function. The cessation of cell division after a series of sub-cultivations is termed replicative senescence (RS). Most current evidence indicates that the molecular mechanisms of cellular ageing are based on telomerase dysfunction and, consequently, telomere loss, which leads to chromosomal instability [34]. Similar changes leading to cellular ageing can also be triggered by a range of stress stimuli, including those that initiate DNA damage [35], oxidative stress [36], or oncogenic activation [37]. Cells exposed to these factors exhibit a premature senescence phenotype at an accelerated rate, likely due to telomerase disruption rather than telomere shortening per se. This reduced cell proliferation is termed “stress-induced premature senescence” (SIPS) [38].

Thus, to investigate the potential mechanisms regulating endothelial senescence, we established two parallel models of cellular senescence: replicative senescence and stress-induced premature senescence. Furthermore, to determine whether the observed changes in modulating the ageing process of endothelial cells are universal across this cell type, we used endothelial cells of different origins: Human Umbilical Vein Endothelial Cells (HUVECs) and Human Microvascular Endothelial Cells (HMEC-1). Although HMEC-1 cells were transfected with the pSVT vector, a pBR-322-based plasmid containing the coding region of the Simian virus 40A gene product for their immortalisation, we observed that long-term culture induced changes in their morphology and function, and the acquisition of a senescence-like phenotype during our handling. It seems that the observed changes in phenotype and inhibited proliferation result from replication stress and mitotic dysfunction, rather than from classical telomere attrition-driven replicative senescence [39].

We observed that prolonged propagation of healthy HMEC-1 cells and HUVECs promoted replicative senescence. Our observations are consistent with previous reports showing that endothelial cell senescence leads to permanent growth arrest via replicative senescence due to cumulative telomere shortening [40]. Furthermore, we observed that oxidative stress induced a senescence-like phenotype in the studied endothelial cell lines. These findings are consistent with previous reports suggesting that endothelial cells undergo SIPS under oxidative stress [41]. In general, we confirmed that the cells of both research models entered the senescent state by observing the appearance of classical features of the senescent phenotype, such as growth retardation, morphological changes reflected by cell enlargement, elevated senescence-associated β-gal (SA-β-gal) activity, and increase in abundance of p16 and p21, which are well-known cyclin-dependent kinase (CDK) inhibitors. Importantly, a significant increase in all these changes has previously been observed in endothelial cells obtained from elderly donors or from patients with age-related vascular disorders [42,43,44]. It should be noted that replicative senescence had twice the impact on cell enlargement as stress-induced senescence. We propose that the greater increase in cell diameter observed in RS cells may result from the gradual nature of replicative ageing, which allows for cytoskeletal remodelling, adhesive and mechanotransductive changes that not only accumulate but are also sustained and cumulated over time. In contrast, H_2_O_2_-induced SIPS is a result of acute stress that can trigger classic senescence markers, but may not result in complete, stable cytoskeleton remodelling. This hypothesis is consistent with reports showing that SIPS does not fully activate the ordered programme of RS [45], that RS and SIPS differ in genes associated with senescent morphogenesis.

Furthermore, we also analysed the markers specific to endothelial senescent cells. We investigated changes in the phosphorylation and expression of endothelial NO synthase (eNOS), which is responsible for nitric oxide (NO) production, and in the expression of intercellular adhesion molecule 1 (ICAM-1), which plays an important role in enhanced monocyte adhesion [25,46]. We revealed dysfunction in NO synthesis, as evidenced by reduced eNOS activity and protein levels in both cellular senescence models. At the same time, we observed increased ICAM-1 levels, which have also been observed during endothelial senescence [46]. Furthermore, it has been revealed that senescent cells develop long and dense vimentin filament networks in contrast to the short and thick ones observed in young cells [47,48]. Notably, in our models of cellular ageing, we also detected senescence-associated alterations in the vimentin cytoskeleton. We found that endothelial cells undergoing replicative and stress-induced premature senescence exhibited impaired function, including a reduced ability to form capillary-like structures, consistent with previous studies demonstrating diminished angiogenic capacity in senescent endothelial cells [49].

Having established a cellular model of endothelial senescence, we investigated whether endothelial ageing involves the integrin signalling pathway, which plays a crucial role in endothelial cell biology. It has previously been proposed that integrins regulate the cellular senescence process [50,51]; since senescence has been associated with remodelling of the focal adhesion complex and ILK is an important component of focal adhesions, we investigated whether ILK is regulated during endothelial senescence. Integrin-linked kinase (ILK) is an integrin-binding cytoplasmic multifunctional protein involved in cell–matrix interaction, and participates in the regulation of multiple cellular processes, including proliferation, survival, apoptosis, differentiation, cell adhesion, angiogenesis, migration, and cancer invasion, as reviewed elsewhere [4]. ILK is broadly expressed in many human cells and tissues and is implicated in both physiological and pathological processes. Importantly, although increasing evidence suggests ILK’s role in cellular senescence and organismal ageing [52], there is less understanding of its role in endothelial senescence.

We investigated ILK status in replicative and stress-induced premature senescence of endothelial cells and found its downregulation in both cellular senescence models. Our results stayed in agreement with the previous report, which showed downregulation of ILK in HAECs that undergo replicative senescence [53]. Although reduced ILK protein levels correlated with a senescent phenotype in endothelial cells, it was unknown whether ILK plays a critical or merely accompanying role in endothelial ageing. Thus, in the next step, we investigated the relationship between ILK levels and alterations in cell senescence using two complementary approaches: ILK silencing in young endothelial cells and ILK overexpression in senescent endothelial cells. Our data show that ILK silencing promotes a senescence-like phenotype, as evidenced by alterations in cellular morphology, upregulation of p16, p21, and ICAM-1, and elevated SA β-gal activity. At the same time, we revealed reduced eNOS activity and its protein levels. Moreover, we detected senescence-associated alterations in intermediate filaments and endothelial dysfunction, as evidenced by reduced angiogenesis ability. Conversely, ILK overexpression in aged ECs attenuated selected senescence-associated changes, as evidenced by reduced expression of p16, p21, and ICAM-1. Moreover, old ECs overexpressing ILK restored eNOS activity and protein levels, attenuated senescence-associated alterations in intermediate filaments, and enhanced angiogenesis ability. However, cell morphology and SA β-gal activity in ILK-overexpressing old endothelial cells were not remarkably altered. Therefore, our data support partial rejuvenation rather than complete reversal of endothelial senescence. Taken together, these findings indicate that ILK modulates selected molecular and functional features of endothelial senescence rather than uniformly reversing all senescence-associated traits.

In summary, it is reasonable to suggest that there is an association between high ILK expression and relative resistance to senescence-associated endothelial dysfunction. This is especially relevant given the critical role of ILK in maintaining NO synthesis and eNOS function, thereby preventing endothelial dysfunction. At the molecular level, ILK has been shown to stabilise the interaction between eNOS and Hsp90 protein, thereby ensuring the coupling of eNOS activity with the production of NO, rather than other oxidative derivatives [26]. At the same time, emerging evidence suggests that increasing NO bioavailability and eNOS activation may promote telomerase activity, thereby delaying endothelial cell senescence [54]. Moreover, our data are complementary to a previous study, which showed an important role of ILK in regulating retinal vascular endothelial proliferation, migration, and angiogenesis [55]. 

On the other hand, a limitation of the present study is that NO production was not directly measured. We assessed eNOS protein levels and Ser1177 phosphorylation, a well-established indicator of eNOS activation [56,57]. Thus, the observed changes support ILK-dependent modulation of the eNOS signalling pathway, although future studies involving direct NO measurements would provide additional functional confirmation, particularly given that phosphorylation-dependent regulation of eNOS does not always directly mirror NO formation in endothelial cells [58].

However, it is essential to note that ILK has also been suggested to stimulate cellular senescence and organismal ageing. Previous studies showed that in non-endothelial cells, such as cardiac fibroblasts, smooth muscle cells, myoblasts, dental pulp stem cells, and renal cells, ILK overexpression induced various cellular senescent phenotypes [8,9,10,57,59]. At the same time, other groups reported the opposite effect of ILK in cancer, where inactivation or repression of ILK induces a senescence phenotype in skin tumours, benign colon adenomas, gastric cancer, and retinoblastoma [58,59]. Thus, the different regulatory functions of ILK in the cellular senescence of normal versus transformed cells appear to be context-dependent.

In conclusion, we demonstrate that integrin-linked kinase is downregulated in both replicative and stress-induced premature senescent endothelial cells. Moreover, our study was the first to reveal that ILK plays a critical role in endothelial ageing. We revealed that ILK silencing induced a senescence phenotype in endothelial cells, whereas ILK overexpression partially rejuvenated senescent endothelial cells and restored their functionality. Since senescence impairs endothelial function and leads to defective vascular repair, a better understanding of ILK’s role in senescence-associated dysfunction may help develop strategies to attenuate the effects of ageing on the vasculature.

## Data Availability

The data presented in this study are available upon request from the corresponding author.

## Supplementary Materials

The following supporting information can be downloaded at https://www.mdpi.com/article/10.3390/cells15121081/s1. Figure S1: Senescence impairs vimentin filaments. Endothelial cells in early and late passages were treated with H_2_O_2_. Then, senescence-associated alterations of vimentin intermediate filaments were estimated by immunostaining. Representative images are shown. Scale bars, 100 μm; Figure S2: ILK silencing impairs vimentin filaments. Endothelial cells at low passages (HMEC-1 at passage no 15, and HUVEC at passage no 1) were transfected with siRNA ILK or scramble siRNA (negative control), and 48 h later, senescence-associated alterations of vimentin intermediate filaments were estimated by immunostaining. Representative images are shown. Scale bars, 100 μm; Figure S3: ILK overexpression restores vimentin filaments. Endothelial cells at late passage (HMEC-1 at passage no 33, and HUVEC at passage no 4) were transfected with pcDNA3.1-ILK or an empty pcDNA3.1 vector (as a control) and, after 2 passages, were subjected to analyses. Senescence-associated alterations of vimentin intermediate filaments were estimated by immunostaining. Representative images are shown. Scale bars, 100 μm.

## Abbreviations

The following abbreviations are used in this manuscript:

EC	Endothelial cell
eNOS	Endothelial Nitric Oxide Synthase
HMEC-1	Human Microvascular Endothelial
HUVEC	Human Umbilical Vein Endothelial Cells
ICAM-1	Intercellular adhesion molecule-1
ILK	Integrin-Linked Kinase
NO	Nitric Oxide
RS	Replicative senescence
SA-β-gal	Senescence-associated β-galactosidase
SIPS	Stress-induced premature senescence
