# Supplementary material for: Integrin-Linked Kinase Plays an Active Role in the Regulation of Endothelial Senescence
Source: Cells. 2026 Jun 14;15(12):1081. doi: 10.3390/cells15121081 (PMC13296395; doi:10.3390/cells15121081)
Supplement: Supplementary file 1 [file cells-15-01081-s001.zip › cells-4317955-supplementary.pdf]

cells

MDPI

Article

Integrin-Linked Kinase Plays an Active Role in The Regulation of Endothelial Senescence

Wojciech M. Ciszewski [1,*], Ewa Macierzyńska-Piotrowska [1], Katarzyna Sobierajska [1]

[1] Department of Molecular Cell Mechanisms, Medical University of Lodz, Mazowiecka 6/8 Str., 92-215 Lodz, Poland; ewa.macierzynska-piotrowska@umed.lodz.pl (E. M-P), katarzyna.sobierajska@umed.lodz.pl (K.S.)

* Correspondence: wojciech.ciszewski@umed.lodz.pl; Tel.: +48-422-725-728

Supplementary data

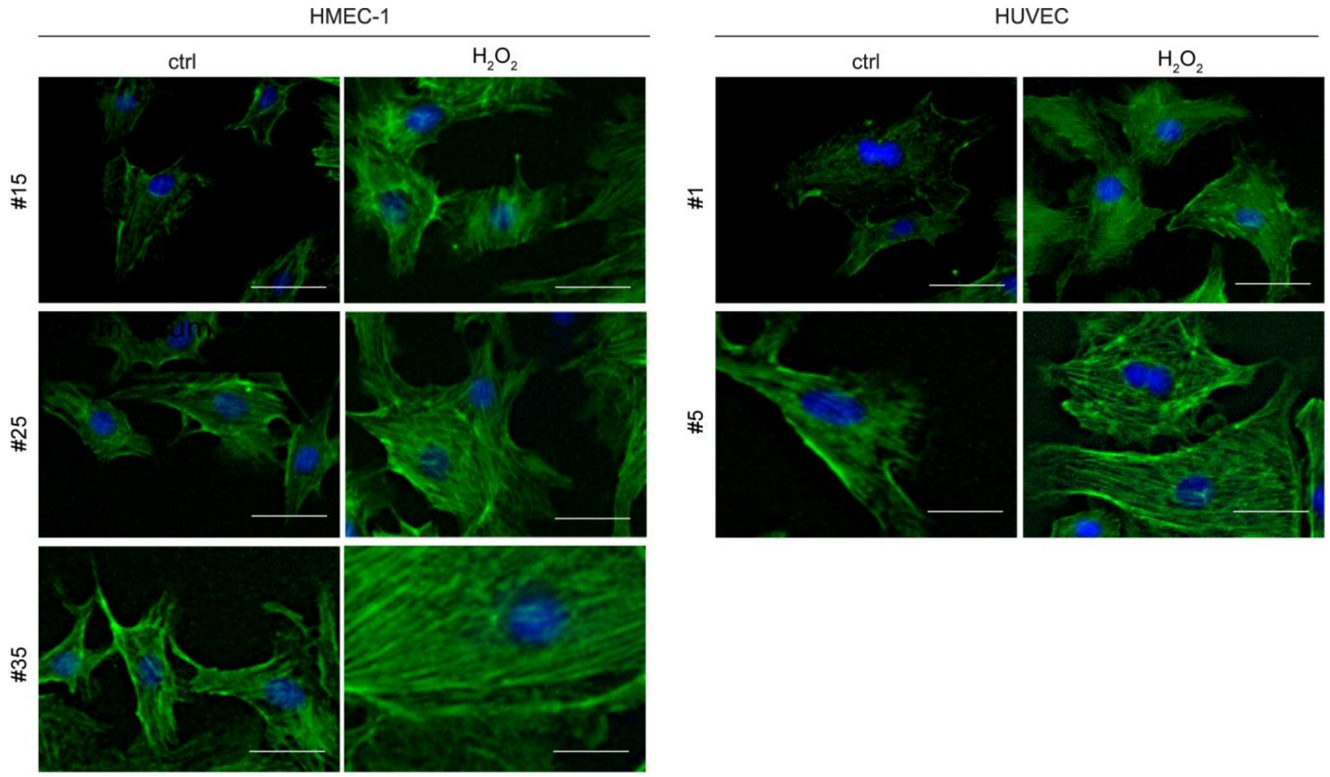

Figure S1. Senescence impairs vimentin filaments. Endothelial cells in early and late passages were treated with H_2O_2. Then, senescence-associated alterations of vimentin intermediate filaments were estimated by immunostaining.. Representative images are shown. Scale bars, 100 µm.

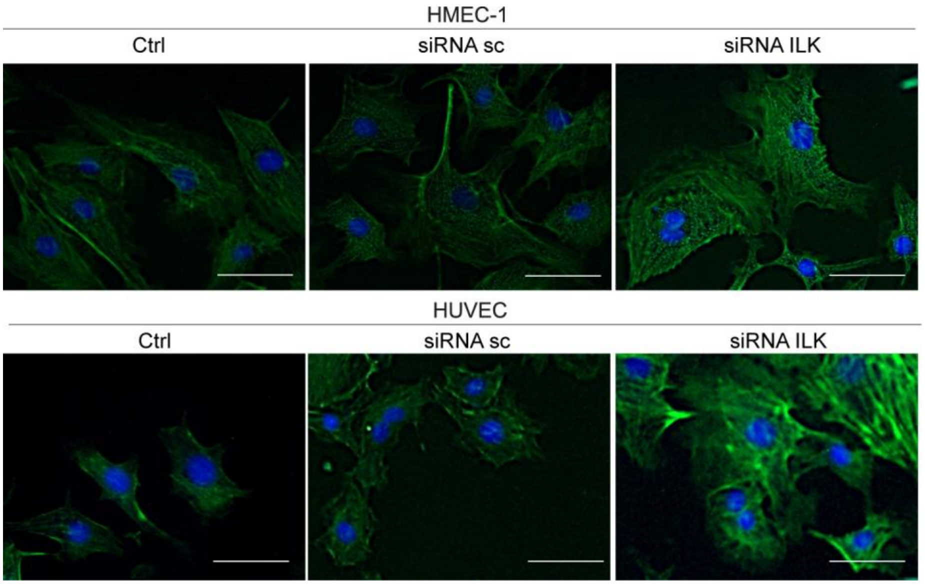

Figure S2. ILK silencing impairs vimentin filaments. Endothelial cells at low passages (HMEC-1 at passage no 15, and HUVEC at passage no 1) were transfected with siRNA ILK or scramble siRNA (negative control), and 48 h later, senescence-associated alterations of vimentin intermediate filaments were estimated by immunostaining. Representative images are shown. Scale bars, 100 μm.

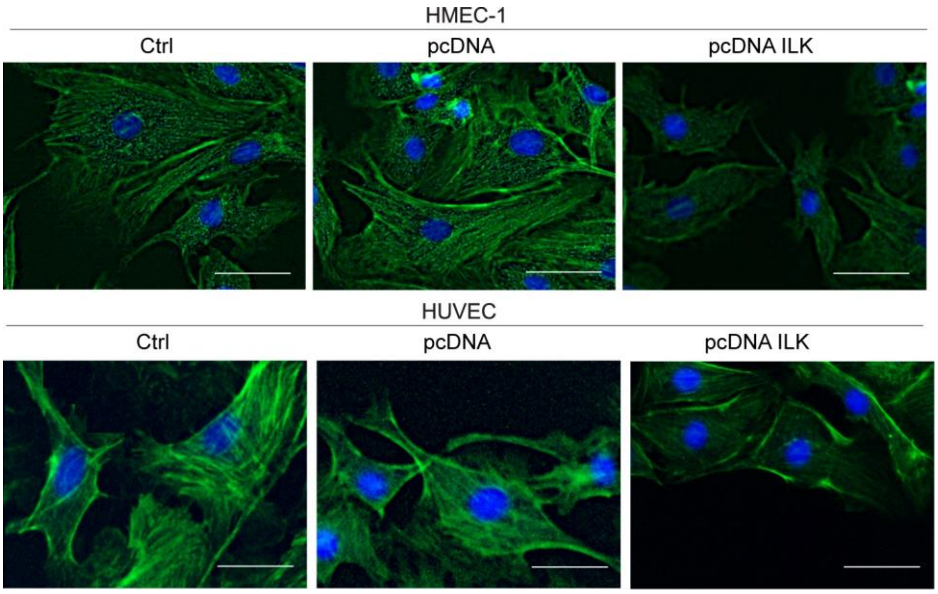

Figure S3. ILK overexpression restores vimentin filaments. Endothelial cells at late passage (HMEC-1 at passage no 33, and HUVEC at passage no 4) were transfected with pcDNA3.1-ILK or an empty pcDNA3.1 vector (as a control) and, after 2 passages, were subjected to analyses. Senescence-associated alterations of vimentin intermediate filaments were estimated by immunostaining. Representative images are shown. Scale bars, 100 μm.
